# Distance-Constraint *k*-Nearest Neighbor Searching in Mobile Sensor Networks

**DOI:** 10.3390/s150818209

**Published:** 2015-07-27

**Authors:** Yongkoo Han, Kisung Park, Jihye Hong, Noor Ulamin, Young-Koo Lee

**Affiliations:** Department of Computer Engineering, Kyung Hee University, Suwon 446-701, Korea; E-Mails: ykhan@khu.ac.kr (Y.H.); pedo24@khu.ac.kr (K.P.); hjhh@khu.ac.kr (J.H.); Noorulamin@khu.ac.kr (N.U.)

**Keywords:** wireless sensor networks, *k*-nearest-neighbors query, spatial queries

## Abstract

The k-Nearest Neighbors (kNN) query is an important spatial query in mobile sensor networks. In this work we extend *k*NN to include a distance constraint, calling it a l-distant k-nearest-neighbors (l-kNN) query, which finds the k sensor nodes nearest to a query point that are also at l or greater distance from each other. The query results indicate the objects nearest to the area of interest that are scattered from each other by at least distance *l*. The l- kNN query can be used in most kNN applications for the case of well distributed query results. To process an l-kNN query, we must discover all sets of kNN sensor nodes and then find all pairs of sensor nodes in each set that are separated by at least a distance l. Given the limited battery and computing power of sensor nodes, this l-kNN query processing is problematically expensive in terms of energy consumption. In this paper, we propose a greedy approach for l- kNN query processing in mobile sensor networks. The key idea of the proposed approach is to divide the search space into subspaces whose all sides are l. By selecting k sensor nodes from the other subspaces near the query point, we guarantee accurate query results for l- kNN. In our experiments, we show that the proposed method exhibits superior performance compared with a post-processing based method using the kNN query in terms of energy efficiency, query latency, and accuracy.

## 1. Introduction

Advancements in wireless technology and sensors have enabled rapid development of mobile sensor networks in which moving sensor nodes are wirelessly connected. Recently, mobile sensor networks have received a lot of attention because they have a variety of applications, such as intelligent transportation systems [1], wildlife conservation systems [2], and battlefield surveillance systems [3].

The k-nearest neighbors (kNN) query is an important spatial query in spatial or multidimensional databases [4,5,6]. In mobile sensor networks, the kNN query is to find the k sensor nodes closest to the query point q. The kNN query can be used in vehicle navigation, wildlife social activity discovery, forest fire impact investigations, and squad/platoon searches on the battlefield [7].

Traditional kNN query processing techniques assume the context that location data are collected in a centralized database [8,9]. Collecting a massive amount of sensed data to a centralized database incurs unnecessary and redundant message transmissions. These traditional techniques are infeasible for mobile sensor networks due to the high communication cost and energy consumption. Recently, in-network techniques have been proposed to overcome these problems. Representative kNN query processing techniques are PT [10], KPT [11], DIKNN [12,13] and GDRKNN [14]. Studies of these methods have shown improved performance by using index and *ad hoc* geographic routing techniques. Especially, they have focused on reducing the number of transmissions because the greatest energy consumption by sensor nodes is due to communication.

This paper focuses on a novel spatial query problem, the kNN query with a distance constraint, named l-distant k-nearest neighbors (l-kNN). The l-kNN query finds the k sensor nodes nearest to the query point q and at least separated from each other by a distance l. One of main objectives of the kNN query is to facilitate collection of sensor data samples around the query location in the applications [15]. However, since the existing kNN query does not consider the distribution of the objects in the query result, it has the severe drawback that the objects sampled can be skewed only in a small area rather than the whole area of interest. If we need well distributed or wide coverage of the kNN result in the applications of the kNN query, the l-kNN query is more suitable because it finds well scattered objects over the area of interest. An example of l-kNN query is survival discovery on the battlefield [7]. Suppose we want to seek for the nearby survivals around a battlefield stronghold. While the kNN query finds simply k nearest survivals, our proposed l-kNN query can reveal the distribution of survivals around the battlefield stronghold.

**Figure 1:**
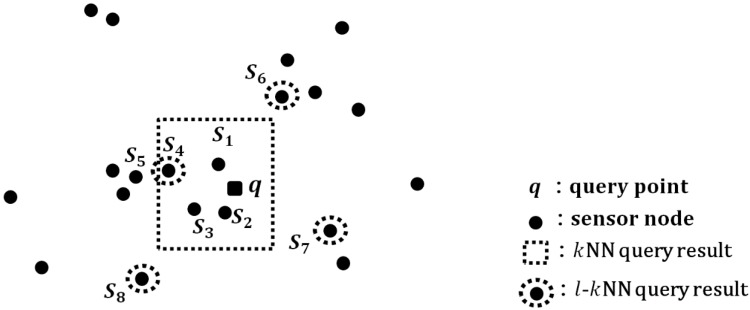
Comparisons of results between the kNN and the l-kNN queries.

Figure 1 shows comparisons of results between the kNN and the l-kNN queries when k is set to 4. Given the query point q, the result for the kNN query is $S_{1}$, $S_{2}$, $S_{3}$ and $S_{4}$, which are skewed in a small area and cover only the left side of the query point. When k increases from 4 to 5, the right side is still not covered because $S_{5}$ is selected additionally. The result for the l-kNN query is $S_{4}$, $S_{6}$, $S_{7}$ and $S_{8}$, which covers the wider area in a well distributed way. 

Considering the limited computing power and batteries of sensor nodes, processing a l-kNN query is problematically expensive in terms of energy consumption in mobile sensor networks. For n sensor nodes, _*n*_$C_{k}$ is the number of cases possible for selecting k sensor nodes. Moreover, we need to verify the l-distant constraint between each pair of sensor nodes _*k*_$C_{2}$ times for each case. To compute the l-kNN query for all the cases, a great number of message transmissions would be required, which would incur huge battery consumption. Especially, sensor nodes located far away from the query point require large battery consumption because they must transmit messages to the query node through several intermediate sensor nodes.

In this paper, we propose a greedy approach for l-kNN query processing in mobile sensor networks. The key idea of the proposed approach is to divide the search space into subspaces whose sides are all l. By selecting k sensor nodes from the other subspaces near the query point, we can obtain accurate query results for l-kNN. The proposed approach has several challenging issues, including estimating the search space, dividing the search space with the l constraint, and traversing between the subspaces. We present efficient solutions for each of these challenges. In our experiments, we show that the proposed method exhibits superior performance compared with a post-processing based method using the kNN query in terms of energy efficiency, query latency, and accuracy.

The rest of the paper is organized as follows: Section 2 reviews the existing kNN query processing techniques. Section 3 presents the proposed l-kNN query in detail. Section 4 reports the performance evaluation through various experiments. Finally, Section 5 provides the conclusions for the paper.

## 2. Related Work

Wireless sensor networks (WSNs) are composed of many devices that sense, store, and transmit data. WSNs have limitations in processing queries because the sensors have limited power supplies, are vulnerable to failure, and have the dynamic property that their availability can vary over time. 

kNN queries retrieve the k sensor nodes nearest to a point of interest indicated by users, called a query point in WSNs. The kNN query can be used to search in border detection and ecological monitoring. kNN queries in WSNs have been actively studied to optimize performance given the above limitations of WSNs. kNN query processing approaches can be categorized as either fixed index or dynamic index. Fixed-index approaches use a stationary structure based on an R-tree or a spanning tree [10]. These approaches have the critical problem of maintaining the index when the sensor nodes are mobile. Dynamic-index approaches propagate the kNN query gradually along itineraries to collect data from sensors in a specific region [11,12,13]. 

The approaches using a fixed-index structure [10] have several problems. The major problem is that index nodes become system bottlenecks because all the query messages are aggregated by index nodes designated cluster heads. Moreover, large numbers of unnecessary hops from the query point are required because all the query messages are routed along the index hierarchy. This overhead causes significant performance degradation in a large WSN. 

To overcome these issues, approaches that do not use the fixed index structure have been proposed [11,12,13]. KPT [11] assumes a location-aware sensor network and estimates a conservative boundary that includes at least k sensor nodes to avoid messages’ flooding the entire network. However, mobile sensors cause problems in this approach because KPT assumes that the nodes are stationary. The reconstruction cost of the tree is considerably large when the sensor nodes are moving. Moreover, the conservative boundary expands dramatically with increasing k. Some sensor nodes may even turn aside from the boundary during tree construction.

The itinerary-based kNN (IKNN) approach [12,13] has been proposed to solve these issues. This approach uses both sequential and parallel itinerary processing approaches instead of the tree structure. Two formats have been proposed for the itinerary in IKNN: spiral and parallel. With the spiral itinerary format, the query dissemination starts at the node closest to the query point and follows an itinerary in the form of a spiral. When the number reaches k, the query dissemination is stopped, and the result is sent back to the originator. With the parallel itinerary format, the query dissemination also starts at the node closest to the query point, but it then follows two parallel itineraries. The query stores the number of nodes that answered the query. Neighbor nodes in the different itineraries sum their counts to calculate the total number of nodes that answered the query. 

The density-aware itinerary-based kNN (DIKNN) approach [13] divides the region of interest into cone-shaped areas centered at the query point. In each area, an itinerary is created along which a kNN query is propagated. Itinerary information exchanges occur when itineraries encounter a sector border. When a KNN query reaches a kNN boundary, the last query node in each sector sends the partial results directly to the source node. Through good estimation of the kNN boundary, DIKNN improves its query latency over IKNN. However, the accuracy of the kNN boundary estimation is critical. Although the kNN approach dynamically adjusts its estimated kNN boundary, redundancy still exists in the kNN query result of DIKNN because the partial kNN query results from all sectors are sent back to the source node without any validation. Furthermore, itinerary structures developed in IKNN and DIKNN do not explore the issue of optimizing the number of kNN query threads. 

kNN boundary estimation methods have been proposed to improve the performance of the kNN query. The grid division routing mechanism based kNN (GDRKNN) [14] controls the query boundary expansion based on the number of kNN sensors in wireless sensor networks of skewness distribution. The extended explosion method (EXP) [16] estimates the kNN boundary based on the density of sensor nodes in the entire sensor network where the density of sensor nodes is not uniform.

Many studies have proposed ways to optimize the performance of the kNN query in various environments or conditions [17,18]. Xie *et al.* [17] proposed kNN query processing method in wireless and robot networks (WSRNs). Huang *at al.* [18] proposed an efficient algorithm to process the kNN query for moving objects in a grid-based sensor network.

However, to the best of our knowledge, no research has been done on kNN processing considering distance constraints between nearest neighbors in WSNs. We focus on searching for the k nearest neighbors that satisfy the constraint that each sensor node is separated away from ever other by at least a distance *l*.

## 3. *l-k*-Nearest-Neighbors Query Processing

In this section, we first provide a formal definition of our l-kNN problem in Section 3.1. We then describe the overall process of l-kNN in Section 3.2 and explain core phases of l-kNN in detail in Section 3.3 and Section 3.4.

### 3.1. Problem Definition

The l- kNN problem is formally defined as follows:

- **Definition 1 (l-distant k-nearest neighbor problem).** Given a set N of sensor nodes, a query point q and valid time V, find a subset N′ of N with k nodes *(i.e.*, $N^{\prime }\subseteq N$, $\left|N^{\prime }\right|$= k) such that at time *V*, $\forall n_{1}\in N^{\prime }$, $\forall n_{2}\in \left(N-N^{\prime }\right)$, $DIST(n_{1},q)\leq DIST(n_{2},q)$ and $\forall n_{3}\in N\prime$, $\forall n_{4}\in N^{\prime }$, $DIST(n_{3},n_{4})\geq l$, where DIST( ) denotes the Euclidean distance function.
- We assume that the sensor nodes are randomly distributed and moving in wireless sensor networks. Each sensor node's movement direction and velocity are randomly determined. All sensor nodes have their own storage and computing power to process queries. Each sensor is aware of its own location via GPS or other localization technique, such as beaconing.
- We define new concepts to traverse the search space for l-kNN.
- **Definition 2 (sector).** Given a query point q, a sector is the fan-shaped area S between two radii centered at q. Given that the number of sectors is n, each sector $S_{i}$ has the same central angle $\frac{2\pi }{n}$. Sector numbers are reassigned in each track.
- **Definition 3 (track).** Given a query point q, a track is the ring-shaped area T centered at q. The i-th track centered at q is defined as presented in Equation (1), where $C_{q}$(r) denotes a circle centered at q with a radius $r_{i}$, where $r_{i}>r_{i-1}$.
  (1) $$T_{i}=\{\begin{array}{c} C_{q}(r_{i}),\;if\;i=1\\ C_{q}\left(r_{i}\right)-C_{q}\left(r_{i-1}\right),\;if\;i>1 \end{array}$$
- **Definition 4 (track-sector).** A track-sector TS is the intersecting area of a track and a sector.
- We define the following notations to simplify the explanation of l-kNN. A *home node* (*H-node*) is the sensor node nearest to the query point q. We call a sensor node selected for query results a *query node* (*Q-node*). If a track contains a query node, it is called a *query track*; otherwise, a *margin track*. If a sector contains a query node, it is called a *query sector*; otherwise, a *margin sector*.

**Figure 2:**
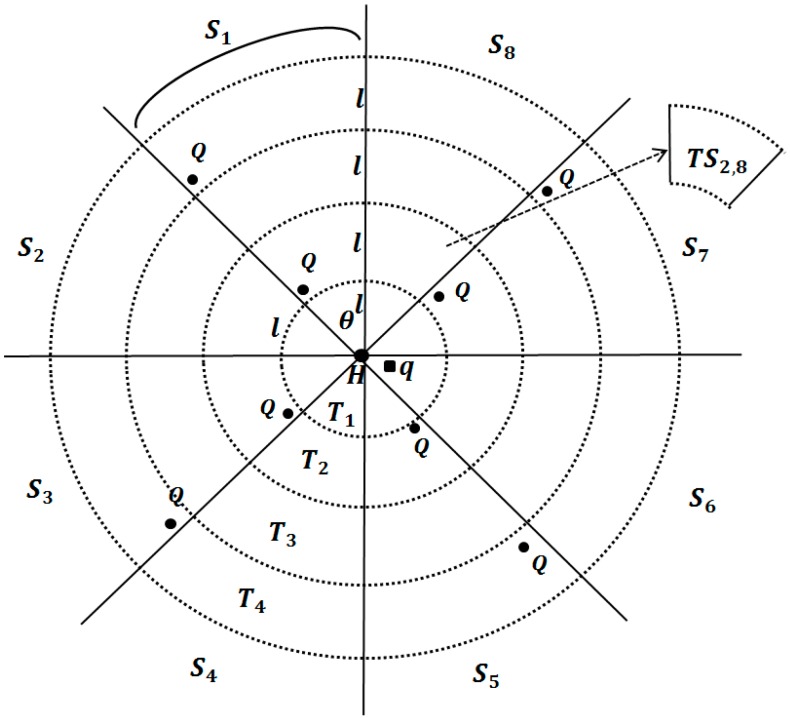
Basic concept for l-kNN query processing.

Figure 2 shows examples of sectors, tracks and track-sectors. $T_{1}$, $T_{2}$, $T_{3}$ and $T_{4}$ are tracks having radii l, 2l, 3l and 4l centered at q, repectively. The number of sectors is eight, and each sector has a central angle $\frac{2\pi }{8}$. The track-sector $TS_{2,8}$ represents the intersecting area of $T_{2}$ and $S_{8}$. $T_{2}$ and $T_{4}$ are query tracks, and the others are margin tracks. We regard $T_{1}$ is a margin track, although the H-node is selected for the query results. $S_{1}$, $S_{3}$, $S_{5}$ and $S_{7}$ are query sectors, and the other sectors are margin sectors.

### 3.2. Overall Process

The basic concept of our l- kNN query processing is to divide the search space into several track-sectors whose sides are all larger than l. We then select one Q-node in each of the other track-sectors. Therefore, the Q-nodes are at least distance l from each other because a track-sector with a Q-node is surrounded by margin tracks and sectors. For example, $TS_{2,1}$ is surrounded by two margin tracks $T_{1}$ and $T_{3}$, and two margin sectors $S_{2}$ and $S_{8}$, as shown in Figure 2. The overall processing of a l- kNN query consists of four phases:

- Routing phase: A query message is routed from the source node to the H-node. GPSR [19] is adopted as the routing algorithm. In this phase, the sensor network information, such as the number of nodes, is collected without the assistance of any infrastructure.
- l- kNN boundary estimation phase: The H-node estimates a searching boundary. The l-kNN boundary is dynamically estimated according to the distance constraint l and the number of neighbors k.
- Query dissemination phase: The H-node disseminates the query message to other sensor nodes within the l- kNN boundary. In order to guarantee the l distance, the query message is propagated to the query tracks and query sectors.
- Aggregation phase: The H-node aggregates the query results. The aggregated query results are transmitted back to the query source by the GPSR.

The routing and aggregation phases are very similar to existing kNN query processing techniques. Compared with kNN query processing, our proposed l- kNN query processing is mainly different in the l- kNN boundary estimation and query dissemination phases. We explain the details of these phases in Section 3.2 and Section 3.3.

### 3.3. l-kNN Boundary Estimation

The H-node disseminates a query message to the other sensor nodes. The query message is progressively propagated to sensor nodes farther from the H-node. Because we do not have information about the sensor node distribution, we must estimate a maximum boundary for the l- kNN query (l-kNNB) by considering the worst case. A large l-kNNB guarantees correct query results but incurs large energy consumption and slow query responses. However, a small l-kNNB decreases query accuracy because some query results are located outside of the l-kNNB.

Let R be the radius of the l-kNNB. According to the definition of an l-kNN query, we need to find the k sensor nodes nearest to the H-node that are at least distance l apart. Therefore, R is calculated as in Equation (2). All query results are then guaranteed to be located within the radius R:

(2) R=k×l

A sensor node can send messages to other nodes within the sensor’s radio range. If it sends messages to other nodes located outside its range, the query message traverses several hops between the source and destination. Therefore, we need to estimate l-kNNB differently according to the ratio of l to the sensor radio range r. We refine Equation (2) as Equation (3) with consideration of the ratio:

(3) $$T_{i}=\{\begin{array}{c} \;k\times r,\;if\;l<r\\ k\times 2\times r,\;if\;l=r\\ k\times \left(\lceil \frac{l}{r}\rceil +1\right)\times r,\;if\;l>r \end{array}$$

If l<r, l-kNNB is estimated as k×r. A single r is enough to maintain the distance l between two Q-nodes because l is shorter than r. If l=r, l-kNNB is estimated as k×2×r. Although l is equal to r, we need 2×r to ensure the distance l is maintained between two Q-nodes. This is because of the possibility that no sensor node exists on the circumference of r. If l>r, l-kNNB is estimated as $k\times \left(\lceil \frac{l}{r}\rceil +1\right)\times r$. To maintain l, we need one more r than for the case of l=r because l is larger than r.

**Figure 3:**
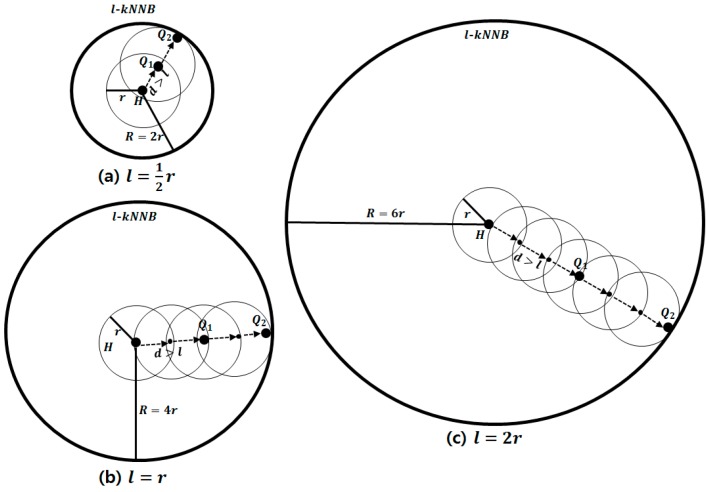
l-kNNBs with various ratios of r to l. (**a**) l-kNNB when r is larger than l; (**b**) l-kNNB when r is equal to l; (**c**) l-kNNB when r is smaller than l.

Figure 3 shows examples of l-kNNBs with various ratios of r to l when k is 2. Figure 3a–c represent estimates of l-kNNBs for the cases of r>l, r=l and r<l, repectively. According to Equation (3), R is estimated as 2×r, 2×2×r and 2×(2+1)×r when l is $\frac{1}{2}r$, r and 2r, respectively. The distance d between the Q-nodes is longer than l in all cases.

### 3.4. l-kNN Query Dissemination

After l-kNNB is determined, the query message is progressively disseminated from the H-node to the circumference of l-kNNB. Tracks are extended from the H-node to l-kNNB, and each track is divided into sectors. The query is propagated to the query tracks and query sectors. This process is repeated until the query message reaches l-kNNB.

We observe that odd track numbers are margin tracks and even track numbers are query tracks. This is because we select the Q-nodes in the other tracks. In each margin track, the central angle $\theta_{i}$ and the number of sectors $n_{i}$ are calculated for dividing the track into sectors. Notice that the central angle and the number of sectors are recalculated in each margin track. This is because this procedure keeps tight distances between query sectors. If we divide all tracks into sectors with the same central angle as in the first track, farther track-sectors have longer chards than l, leading to low quality query results because the size of margin sectors is determined too loosely.

Consider an H-node with coordinates H(a,b) and any margin track $T_{i}$ centered at H with radius l. $T_{i}$ is sequentially divided into several sectors from the baseline y = b in the counterclockwise direction. The central angle $\theta_{i}$ and the number of sectors $n_{i}$ are determined depending on the distance constraint l. We have a well-known equation involving a central sector, a radius, and a chord as in Equation (4):

(4) $$radius\times sin\left(\frac{\theta }{2}\right)=\frac{1}{2}\times chord$$

Because we want all sides of each track sector to have length l, $\theta_{i}$ is calculated as $\left(l\times i\right)\times sin\left(\frac{\theta_{i}}{2}\right)=\frac{1}{2}\times l$. We can also calculate $n_{i}$ as $n_{i}=\frac{2\pi }{\theta_{i}}$. When a track is divided into sectors in the counterclockwise direction, the last sector has a central angle smaller than θ. In this case, the last sector is merged with the previous sector. In this way, we can guarantee that all chords of sectors have lengths of at least l.

In each query track, Q-nodes are selected in every other track-sector. In a track-sector, a length of an upper chord is longer than l because that of a lower chord is l. Therefore, the Q-node is selected around the inner corner intersecting the lower arc and the margin sector in order to keep the l distance constraint tight. In a track-sector of a margin track, an upper chord is l, and a lower chord is shorter than l. This is why we select the Q-nodes in query tracks rather than in margin tracks.

For a tight distance between query tracks and query sectors, it is also possible to set the radius of query tracks and the chords of query sectors to r rather than l, when r is shorter than l. If we set the radii of query and margin tracks to the same distance l, the average distance between Q-nodes is 2×l. By using r for the radii of query tracks and chords of query sectors, the distance is reduced by 2×l to r+l.

**Figure 4:**
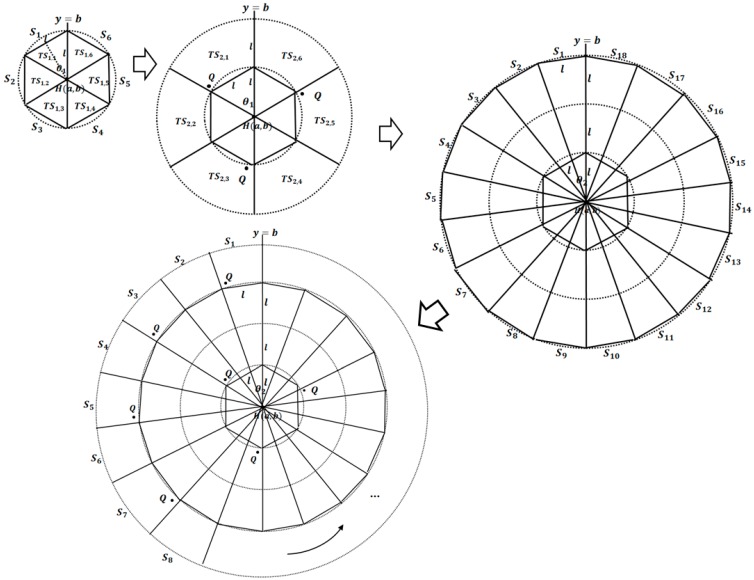
Dividing the search space into tracks and sectors.

Figure 4 shows an example for calculating the number of sectors. $\theta_{1}$ is calculated as $l\times sin\left(\frac{\theta_{1}}{2}\right)=\frac{l}{2}$, and $n_{1}=\frac{2\pi }{\theta_{1}}$, in the first track. In the third track, $\theta_{2}$ is calculated again as $\left(l\times 3\right)\times sin\left(\frac{\theta_{2}}{2}\right)=\frac{l}{2}$ , where $\theta_{1}>\theta_{2}$. The second and fourth tracks are query tracks. Q-nodes are then selected in $TS_{2,1}$, $TS_{2,3}$, $TS_{2,5}$, $TS_{4,1}$, $TS_{4,3}$, $TS_{4,5}$, $TS_{4,7}$, and so on.

In the following, we explain how the query message is disseminated. In a kNN problem, the query message should be disseminated to all sensor nodes in each track-sector because there can be more than one Q-node in a track-sector. However, in our l-kNN problem, we do not need to traverse all sensor nodes. One track-sector can contain at most one Q-node, and we can calculate roughly where the Q-nodes are. Therefore, once the query message reaches a Q-node in a track-sector, the query message escapes this track-sector and heads quickly to the next track-sector containing a Q-node.

The query message traverses between only those track-sectors containing Q-nodes. As explained above, the Q-node is selected around the inner corner intersecting the lower arc and the margin sector in a query track. We regard the corners as virtual vertices because we cannot know whether any sensor nodes exist in the corners. The query message is propagated to the virtual vertex along borders of tracks and sectors by itinerary traversal [7]. The Q-node is selected near the virtual vertex. The itinerary traversal selects the next sensor nodes in the range of $\frac{\sqrt{3}}{2}r$ rather than r in order to balance query accuracy and energy efficiency [20]. If there is no sensor node near the virtual vertex, we seek the Q-node by routing around the virtual vertex based on the right-hand rule of GPSR.

**Figure 5:**
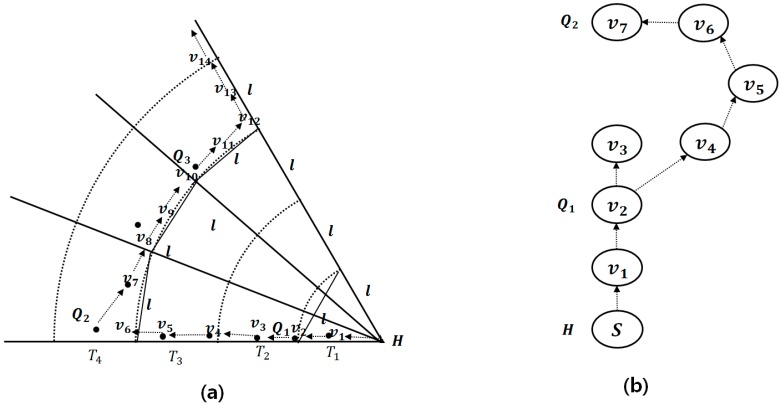
Query dissemination among track-sectors. (**a**) Query dissemination; (**b**) Right-hand rule of GPSR.

Figure 5a shows an example of query dissemination in a sector, where $v_{i}$ is a virtual vertex and $Q_{j}$ is a Q-node. The virtual vertices $v_{2}$, $v_{6}$ and $v_{10}$ are the corners intersecting the lower arc and the margin sector in query tracks. Q-nodes $Q_{1}$, $Q_{2}$ and $Q_{3}$ are the nearest vertices to the virtual vertices, respectively. The query message is propagated from $Q_{1}$ to $v_{6}$ and from $Q_{2}$ to $v_{10}$ along the borders of the sector and tracks by itinerary traversal. Figure 5b shows an example of the right-hand rule of GPSR. If there is no sensor node near the virtual vertex $v_{3}$, we try to route around $v_{3}$ to seek the path from H-node to the next Q-node, $Q_{2}$.

For fast query processing, we adopt parallel computing for query dissemination in each sector. The query dissemination in each sector is an independent subtask. Therefore, this parallelization reduces query latency without accuracy degradation. The number of subtasks has a great effect on the performance. When the number of subtasks is large, network throughput degrades because contentions and collisions occur frequently at the data link and physical layers. Conversely, a small number of subtasks increases latency. Considering this analysis, we determine the number of subtasks as the number $n_{1}\;$of sectors in the first track. Since the query message traverses only the track-sectors containing Q-nodes that are surrounded by margin tracks and sectors, contentions and collisions are minimized in the parallel query dissemination.

At the end of dissemination, query responses are aggregated at the H-node. The H-node can take more than k number of Q-nodes because l-kNNB is the estimated boundary considering the worst case. To select the nearest k number of Q-nodes, the Q-nodes are sorted in ascending order according to their distances from the H-node. Finally, the H-node selects the top-k number of Q-nodes and transmits back to the query source by GPSR.

### 3.5. Algorithm for Query Dissemination

Algorithm 1 is the pseudo code for processing the l-kNN query. The algorithm searches an H-node h nearest to the given query point q (line 1). The H-node h is included in a query result set (line 3). Flag f is used to check whether any Q-node is selected in a query sector (line 4). The query message is propagated forward from the H-node h to other sensor nodes within the l-kNNB (line 5). *search_next_vnode( )* is called to determine the next forward sensor node (line 6). We explain *search_next_vnode( )* in Algorithm 2, as follows. If the *next_vnode* is the starting point of a new track-sector, the flag f is set to “false” in order to select a Q-node in this track-sector (lines 7–8). The algorithm selects the next forward sensor node nearest to the *next_vnode* by using GPSR (line 9). Lines 6–9 are repeated until reaching l-kNNB. The Q-nodes in the resulting set Q are sorted in ascending order according to their distances from the H-node (line 10). The top- k sensor nodes in Q are returned as the query result (lines 11–12).
**Algorithm 1**
l-k-NN Query Processing QueryDissemination (N, q, l, k, b)**⦁Input:** number of sectors N, query point q, distance constraint l, number of nearest neighbors k, and l-k-NNB b**⦁Output:** a result set including Q-nodes Q;
1:Search a home node h nearest to the query point q;2:*cur_node* ←h;3:Initialize a result set Q←{h};4:flag f ← *false*; // the status that the Q-node is not selected yet in a query sector5:**while** (cur_radius < *b*)6:   *next_vnode* ← search_next_vnode(h, l, k, f, Q, cur_node); // find the next virtual node7:   **if** ( *next_vnode* is the vnode intersecting track and sector) // the starting search point of TS8:    f ← *false*;9:   *cur_node* ← GPSR (*cur_node, next_vnode*); // find the sensor node nearest to the next_vnode10:Sort Q-nodes in Q in ascending order according to the distance from the H-node;11:Q← select top-k Q-nodes;12:**return** Q;

Algorithm 2 is the pseudo code for determining the next search direction. To determine the direction, each sensor node should know its own sector number and track number, and the four borderlines of the track-sector. Algorithm 2 includes the calculation of all of this information about the current sensor node. Each sensor node can calculate the discriminants determining the borderlines of its own track-sector by using its own coordinates and those of the H-node.

The current sensor node calculates its distance from the H-node. The track index that the sensor node belongs to is calculated by dividing the distance by the track radius l (line 2). The algorithm calculates the radius R of the current track by multiplying the track index by the track radius l, which is the upper arc of the current track (line 3). The lower arc of the current track is R- l because the radii of the tracks increase by l. Therefore, we can derive the discriminants that calculate the upper and lower arcs of the track having radius R centered at h (lines 4–5). 

The algorithm checks whether the current track is a margin track or a query track (line 6). If the current track is a margin track, the query message is passed to the next track (lines 11–12). Otherwise, the algorithm checks whether a Q-node is selected (line 7). If a Q-node is selected, the query message is passed to the next track-sector (lines 7–8). Otherwise, the query message is passed to the *next_vnode* along the lower arc in the current track-sector (lines 9–10). The chord is calculated as $\frac{\sqrt{3}}{2}r$ for the central angle $\theta_{v}$. This is the optimal chord to select the next sensor node without interference among the sensor nodes. 

After obtaining $\theta_{v}$ for dividing the search space into sectors, the algorithm calculates the sector number $s_{idx}$ of the current sensor node by using the coordinates of the current sensor node and the home node (lines 13–14). The algorithm derives the discriminants that calculate the right- and left-side lines of the current track-sector by using $s_{idx}$ and $\theta_{v}$ (lines 15–16).

We can obtain the corner points from the four discriminants of the borderlines of the current track sector (line 17). If the current track-sector is a query one and no Q-node is selected, the algorithm selects the sensor node nearest to the corner intersecting the lower arc and the margin sector (lines 18–21).

After selecting the Q-node, the algorithm determines the next direction and the *next_vnode* (lines 22–23). Because the algorithm adopts itinerary traversal [13] to select the next track in a sector, the query message is passed to the next sensor node in the east direction in odd-number tracks. Conversely, the query message is passed in the west direction in even-number tracks. The algorithm finally returns the *next_vnode* as the coordinates of the corner of the next track-sector (line 24).

Algorithm 3 determines the next forward direction of the query message from the current sensor node. According to the itinerary traversal algorithm, the forward direction is determined to be east if the current track index is an odd number, and west if even (lines 1–8). If the current track-sector is the last sector or the margin track, the forward direction is determined to be north because the algorithm does not need to select the Q-node (line 9).
**Algorithm 2** Determining the Next Search Direction  search_next_vnode (h, l, k, f, Q, c)
**⦁Input:** the home node h(x,y), the distance constraint l, the number constraint k, a status flag f, the query result set Q, the current sensor node c(x,y)**⦁Output:** the coordinates of the next virtual node1:Initialize a track-sector discriminants set of current node D←{∅}; /* find four discriminants determining border lines of a track-sector */2:$t_{idx}\leftarrow$ ⌈ $\left(\sqrt{{\left(c.x-h.x\right)}^{2}+{\left(c.y-h.y\right)}^{2}}\;/\;l\right)$⌉ ; // calculate the track index of cur_node by using h and c3:$R\leftarrow l\times t_{idx};$ // calculate the current track radius4:$D\leftarrow D\cup^{\text{​}}\left\{{\left(x-h.x\right)}^{2}+{\left(y-h.y\right)}^{2}=R_{\text{​}}^{2}\right\}$; // a upper arc of the track-sector5:$D\leftarrow D\cup^{\text{​}}\{{\left(x-h.x\right)}^{2}+{\left(y-h.y\right)}^{2}={\left(R_{\text{​}}-l\right)}^{2}$}; // a lower arc of the track-sector6:**if** ( $t_{idx}$ is an even number ) // if the current track is a query track7:   **if** (f is *true*) // if a Q-node is selected8:    $\theta_{v}\leftarrow$ calculate the angle of a tractor-sector by $R\times \sin\left(\frac{\theta_{v}}{2}\right)=\frac{l}{2}$ ;    // move to the next track-sector9:   **else** // if a Q-node is not selected10:    $\;\theta_{v}\leftarrow \;$calculate the angle for the next vnode by $R\times \sin\left(\frac{\theta_{v}}{2}\right)=\frac{1}{2}\times \frac{\sqrt{3}}{2}r$;    // move to the next vnode11:**else** // if current track is a margin track12:  $\;\theta_{v}\leftarrow l\times \sin\left(\frac{\theta_{v}}{2}\right)=\frac{l}{2}$*;* // move to the next query track13:$\;\theta_{cur}\leftarrow \;$ calculate the angle between the baseline and the current node by $\text{t}an\theta_{cur}=\frac{\text{c}.\text{y}-\text{h}.\text{y}}{\text{c}.\text{x}-\text{h}.\text{x}}$;14:$s_{idx}\leftarrow \;$calculate the sector index of current node by ⌈$\left(\theta_{cur}\;/\;\theta_{v}\right)$⌉ ;15:$D\leftarrow D\cup^{\text{​}}\left\{y=tan\left(\theta_{v}\times s_{idx}\right)\left(x-c.x\right)+c.y\right\}$; // a left-side line of the track-sector16:$D\leftarrow D\cup^{\text{​}}\left\{y=tan\left(\theta_{v}\times \left(s_{idx}-1\right)\right)\left(x-c.x\right)+c.y\right\}$; // a right-side line of the track-sector17:Calculate intersecting points P={ne, nw, se, sw} between each pair of the track-sector discriminants, such as the upper arc n, the lower arc s, the left-side line w, and the right-side line e;/* check that whether the Q-node is selected or not in this tracksector */18:**if** (IsSelectTrackSector ($t_{idx},s_{idx}$) && f is false)19:  Find the sensor node n nearest to the corner intersecting the lower arc and the margin sector;20:  $\;Q\leftarrow Q\cup^{\text{​}}n$;21:   f←true;22*:**dir*  ← getNextDirection($t_{idx},\;s_{idx}$); // decide the next direction by using the track and sector indexes23:*next_vnode*  ← select the next moving point in P={ne, nw, se, sw} according to *dir*;24*:****return** next_vnode*;
**Algorithm 3** Determining the Next Forward Direction getNextDirection ($t_{idx},\;s_{idx}$)
**⦁Input:** a track index $t_{idx}$, a sector index$\;s_{idx}$**⦁Output:** the direction of the next virtual node
1:$N_{s}\leftarrow$ calculate the total number of partial sectors in the current sector2:**if** ($t_{idx}$%4 == 3) // query track3:   **if** ($s_{idx}<$ N) **return** southeast;4:   **else return** northeast;5:**else if** ($t_{idx}$%4 == 1) // query track6:   **if** ($s_{idx}>1$) **return** southwest;7:   **else return** northwest;8:**else if** ($t_{idx}$%4 == 2) **return** northwest; // margin track9:**else return** northeast; // margin track

Algorithm 4 checks whether the current track-sector $TS_{t,s}$ is a query sector or a margin sector. If the track-sector has an odd-number sector, it is a query sector (lines 1–9). However, if the total number of sectors is an odd number and the current track-sector is the last sector, it is determined to be a margin sector because two consecutive sectors that are the first and the last sectors cannot be query sectors (lines 3–5).
**Algorithm 4** Determining the Sector TypeIsSelectTrackSector ($t_{idx},s_{idx}$)⦁**Input:** a track index $t_{idx}$, a sector index $s_{idx}$ ⦁**Output:** a Boolean value representing whether this TS is in a query sector or in a margin sector1: $N_{t}\leftarrow$calculate the total number of tracks 2: $N_{s}\leftarrow$calculate the total number of sectors in the current track 3: **if (**$N_{s}$ is an odd number) 4:   **if** ($s_{idx}$ is an odd number & $s_{idx}$
≠
$N_{s}$) 5:    return true; //current track-sector is in a query sector 6: **else** 7:   **if** ($s_{idx}$ is an odd number) 8:    return true; //current track-sector is in a query sector 9: **return** false; //current track-sector is in a margin sector

## 4. Performance Evaluation

In this section, we evaluate the performance of the proposed method in terms of energy efficiency, query latency, and query accuracy. We use the ns-2 simulator [21] for our experiments. We observe the impact of the constraint parameters, such as l and k, on the performance.

### 4.1. Experimental Settings

For the performance evaluation, we compare the proposed method with a post processing based method using the kNN query. The post processing method collects all sensor nodes within the l-kNNB using the DIKNN method [13] and selects k number of l-distant sensor nodes from the collected sensor nodes. We call the post processing method *DIKNN* in our experiments.

The proposed method and DIKNN are implemented based on the ns-2 simulator. For the routing protocol in sensor networks, we use the geo-routing protocol GPSR [19] in the ns-2. The sensor nodes are randomly distributed in the wireless sensor networks. The movement pattern of the sensor nodes is represented by the random waypoint model (RWP). Each sensor moves to the arbitrary destination at a random speed ranging from 0 to 20. We fix the network size at 300 × 300 ${\text{m}}^{2}$. We vary the number of sensor nodes from 300 to 1000, and the node degree (*i.e.*, neighbor count of each sensor node) ranges from 5 to 20. The distance between the source node and the home node for each query is set to 250 m. The query response size of each sensor nodes is 10 bytes. The performances are obtained by averaging the result over 10 simulation runs. Table 1 summarizes the datasets used for the experiments.

**Table 1:** Summary of datasets.

Name	Network Size	Number of Nodes
D-300	300 × 300 ${\text{m}}^{2}$	300
D-500	300 × 300 ${\text{m}}^{2}$	500
D-1000	300 × 300 ${\text{m}}^{2}$	1000

Query latency is measured as the elapsed time between when the query is issued by the query point and when query responses are returned. Energy consumption is measured as the total amount of consumed energy during the query processing in the wireless sensor networks. Query accuracy is measured as the set similarity of distance between the ground truth and the proposed method. We use the Hausdorff distance algorithm [22] for the set similarity. Given two result sets of l-kNN, $A=\left\{a_{1},\;a_{2},\ldots ,a_{m}\right\}$ and $B=\left\{b_{1},\;b_{2},\ldots ,b_{n}\right\}$, the Hausdorff distance calculates set similarity as in Equation (5), where $a_{i}$ is the distance from sensor node $s_{i}$ to the home node:

(5) $$H\left(A,B\right)=\frac{1}{m\;}\underset{a\in A}{\sum }\underset{b\in B}{min}\left|\left|a-b\right|\right|$$

### 4.2. Experimental Results

**Figure 6:**
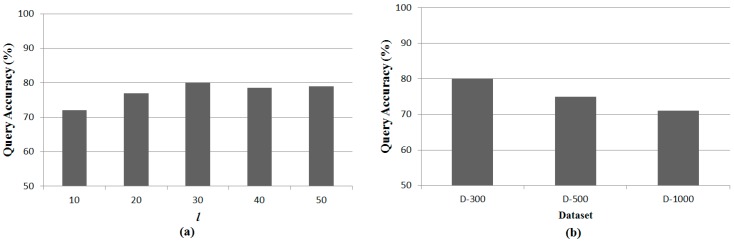
Comparisons of the l**-**kNN accuracy. (**a**) l**-**kNN accuracy with various values of l; (**b**) l**-**kNN accuracy with various datasets.

Figure 6 shows the results for the l-kNN query accuracy compared with the ground truth. The left figure shows the accuracy with various l values, such as 10 m, 20 m, 30 m, 40 m and 50 m in the D-300 dataset when k is set to 30. The l-kNN achieves the highest query accuracy when l is set to 30 m. This is because our method is a greedy algorithm. A small l generates a large number of query result sets because many sensor nodes satisfy the condition of being l distances apart. Therefore, the selected result set has a high probability of being different from the ground truth. In the case of a large l, only a small number of result sets are possible. However, the accuracy decreases because the large l increases the width of each track-sector.

The right figure shows the results for the query accuracy with various datasets, such as D-300, D-500, and D-1000 when l and k are set to 30 m and 30, respectively. As the number of total sensor nodes increases, the query accuracy decreases. This result is related to the densities of the datasets. A dataset with high density includes a large number of result sets satisfying l-kNN. Therefore, the selected result set has a high probability of being different from the ground truth.

**Figure 7:**
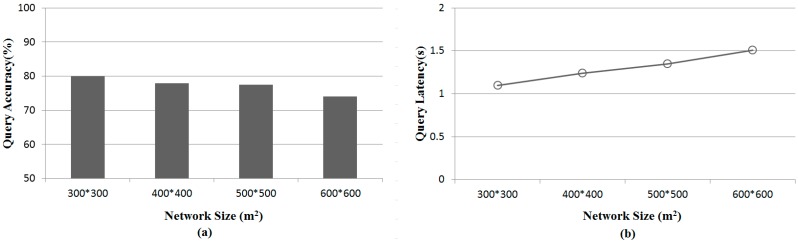
Comparisons of query performance with various network sizes. (**a**) Query accuracy with various network sizes; (**b**) Query latency with various network sizes.

Figure 7 shows the results for the query accuracy and latency with various network sizes when the number of nodes, l and k are 300, 30 m and 30, respectively. As a network size increases, the query accuracy decreases, but the query latency increases. This performance degradation is because the larger network has sparser regions, where a sparse region has a few number of sensor nodes. Therefore, the *l-k*NN algorithm selects sensor nodes whose distance is larger than the ground truth. Similarly, the query latency shows a similar trend to the query accuracy because the average number of transmissions increases in the large network. 

**Figure 8:**
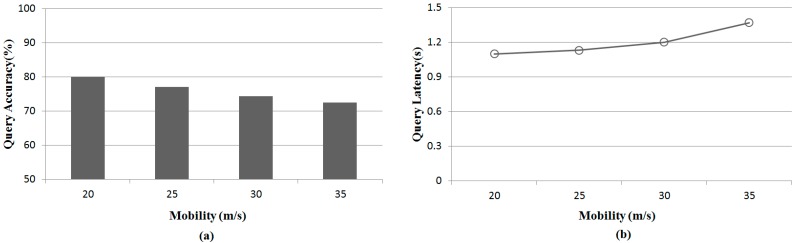
Comparisons of query performance with various values of mobility. (**a**) Query accuracy with with various values of mobility; (**b**) Query latency with various values of mobility.

Figure 8 shows the results of query accuracy and latency with various values of mobility for each sensor in D-300. This experiment for the impact of moving speeds is evaluated by varying maximum moving speeds from 20 m/s to 35 m/s when l and k are set to 30 m and 30, respectively. The left figure shows the query accuracy. As the moving speed increases, the query accuracy decreases. When the moving speed is faster, the selected sensor has a larger distance compared with the ground truth. This is because the selected Q-nodes moves to arbitrary points fast during next Q-nodes are selected. The right figure shows the query latency. The query latency increases rapidly when the moving speed is faster than 30 m/s. This is because the average number of transmissions increases when the moving speed is fast.

**Figure 9:**
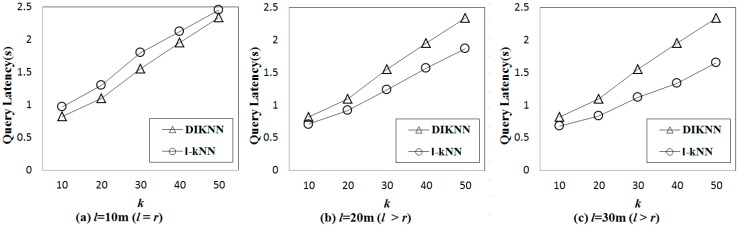
Comparisons of the query latency with various values of l and k. (**a**) Query latency when l is 10 m; (**b**) Query latency when l is 20 m; (**c**) Query latency when l is 30 m.

Figure 9 shows the results of query latency between DIKNN and l-kNN for D-300. When the constraint parameters l and k are sufficiently large, the l-kNN obtains better performance than the DIKNN. When l is smaller than or equal to r (l = 10 m), l-kNN is slower than DIKNN. This is because DIKNN broadcasts only one time to all sensor nodes within the sensor radio range, but l-kNN broadcasts r/l times. When l is larger than r (l = 20 m and l = 30 m), l-kNN is faster than DIKNN because DIKNN each time broadcasts, but l-kNN disseminates the query message to a small number of sensor nodes with GPSR in the l-kNNB. For large enough k, l-kNN is faster than DIKNN when l is larger than r. This explanation is similar to that for the results of l. A large k leads DIKNN to perform many broadcasts. 

**Figure 10:**
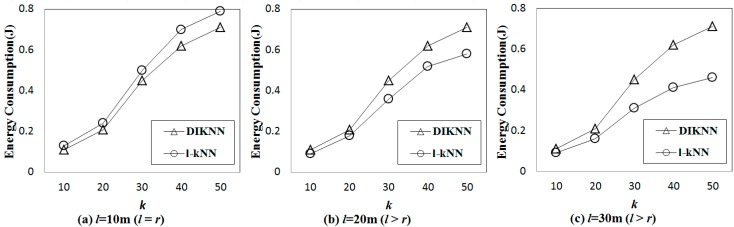
Comparisons of energy consumption with various values of l and k. (**a**) Energy consumption when l is 10 m; (**b**) Energy consumption when l is 20 m; (**c**) Energy consumption when l is 30 m.

Figure 10 shows the results for the energy consumption of l-kNN with various values of l and k for D-300. When l is equal to r, DIKNN consumes less energy than l-kNN. However, when l is larger than r, DIKNN consumes more energy than DIKNN. For large enough k, DIKNN consumes more energy than l-kNN when l is larger than r. These results occur for similar reasons as for the query latency results. When l is larger than r, DIKNN disseminates the query messages to more sensor nodes than l-kNN does, and *vice versa*.

## 5. Conclusions

In this paper, we proposed a solution, the l- kNN, for the novel spatial query problem in mobile sensor networks. The query result implies well scattered objects nearest to the area of interest. The l- kNN approach can be used in most kNN applications if we want to get well distributed or wide coverage of the kNN result. l- kNN divides the search space into several track-sectors in which all sides are equal to or larger than the distance constraint l. By selecting Q-nodes in alternating track-sectors, we have guaranteed l distances between any two Q-nodes. To maintain the l distance tightly, we adjusted the central angles and radii of the track-sectors. We also adopted parallel computing for query dissemination to reduce query latency. Through comprehensive experiments, we showed that the proposed algorithm exhibits superior performance compared with a post-processing based method using the kNN query in terms of energy efficiency, query latency, and accuracy.

## References

[1] United States Department of Transportation Intelligent Transportation System Joint Program Office Home. http://www.its.dot.gov.

[2] Juang P., Oki H., Wang Y., Martonosi M., Peh L., Rubenstein D. Energy-Efficient Computing for Wildlife Tracking: Design Tradeoffs and Early Experiences with Zebranet. Proceeding of 10th International Conference of Architectural Support for Programming Languages and Operating Systems.

[3] Federation of American Scientists Remote Battlefield Sensor System (Rembass). http://fas.org/man/dod-101/sys/land/rembass.htm.

[4] Chon A.A.H.D., Agrawal D. (2003). Range and KNN Query Processing for Moving Objects in Grid Model. Mob. Netw. Appl..

[5] Chen M.-S., Yu P.S., Wu K.-L. (2003). Optimizing Index Allocation for Sequential Data Broadcasting in Wireless Mobile Computing. IEEE Trans. Knowl. Data Eng..

[6] Lee W., Zheng B. DSI: A Fully Distributed Spatial Index for Location-Based Wireless Broadcast Services. Proceedings of International Conference on Distributed Computing Systems.

[7] Wu S.-H., Chuang K.-T., Chen C.-M. (2008). Toward the Optimal Itinerary-Based KNN Query Processing in Mobile Sensor Networks. IEEE Trans. Knowl. Data Eng..

[8] Hjaltason G., Samet H. (1999). Distance Browsing in Spatial Databases. ACM Trans. Database Syst..

[9] Mokbel M., Xiong X., Aref W. SINA: Scalable Incremental Processing of Continuous Queries in Spatio-Temporal Databases. Proceedings of the ACM SIGMOD International Conference on Management of Data.

[10] Demirbas M., Ferhatosmanoglu H. Peer-to-peer Spatial Queries in Sensor Networks. Proceedings of the IEEE International Conference on Peer-to-Peer Computing.

[11] Winter J., Lee W. KPT: A Dynamic KNN Query Processing Algorithm for Location-Aware Sensor Networks. Proceedings of the ACM International Workshop on Data Management for Sensor Network.

[12] Xu Y., Fu T.Y., Lee W.C., Winter J. (2007). Processing K Nearest Neighbor Queries in Location-aware Sensor Networks. Signal Process..

[13] Wu S.-H., Chuang K.-T., Chen C.-M., Chen M.-S. DIKNN: An Itinerary-Based KNN Query Processing Algorithm for Mobile Sensor Networks. Proceedings of the IEEE International Conference on Data Engineering.

[14] Han Y., Tang J., Zhou Z., Xiao M., Sun L., Wang Q. (2014). Novel itinerary-based KNN query algorithm leveraging grid division routing in wireless sensor networks of skewness distribution. Pers. Ubiquitous Comput..

[15] Fu T., Peng W.-C., Lee W.-C. (2010). Parallelizing Itinerary-Based KNN Query Processing in Wireless Sensor Networks. IEEE Trans. Knowl. Data Eng..

[16] Komai Y., Sasaki Y., Hara T., Nishio S. (2014). k-nearest neighbor search based on node density in MANETs. Mob. Inf. Syst..

[17] Xie W., Li X., Narasimhan V., Nayak A. (2014). K Nearest Neighbour Query Processing in Wireless Sensor and Robot Networks. Lect. Notes Comput. Sci..

[18] Huang Y.-K. (2014). Processing KNN Queries in Grid-Based Sensor Networks. Algorithms.

[19] Karp B., Kung H. GPSR: Greedy perimeter stateless routing for wireless networks. Proceeding of the 6th annual international conference on Mobile computing and networking.

[20] Xu Y., Lee W., Xu J., Mitchell G. Processing window queries in wireless sensor networks. Proceedings of International conference of Data Engineering.

[21] The Network Simulator. http://www.isi.edu/nsnam/ns.

[22] Dubuisson M., Jain A.K. A Modified Hausdorff Distance for Object Matching. Proceedings of International Conference on Pattern Recognition.

